# Endovascular stent graft repair of aortogastric fistula caused by peptic ulcer after esophagectomy: A case report

**DOI:** 10.1097/MD.0000000000008959

**Published:** 2017-12-15

**Authors:** Xiao-Qing Wei, Lei Song, Xue-Song Zhang, Kui-Yang Wang, Jie Wu

**Affiliations:** Interventional Therapy Department, The Second Hospital of Dalian Medical University, Dalian, China.

**Keywords:** aortogastric fistula, endovascular stent graft repair, esophagectomy, gastric tube ulceration

## Abstract

**Rationale:**

Aortogastric fistula (AGF) is a rare but devastating clinical complication after esophagectomy. In a recent report, nearly all AGF patients died of massive hemorrhage or aspiration of massive hematemesis. Therefore, timely appropriate treatment of AGF remains a challenge.Herein, we report a case of AGF that resulted from peptic ulceration after esophagectomy and was successfully treated with endovascular stent graft placement.

**Patient concerns:**

A 59-year-old man had undergone video-assisted thoracoscopic esophagectomy for squamous cell carcinoma and esophageal reconstruction using a gastric tube 14 months previously. He suddenly experienced massive hematemesis and unstable circulatory dynamics, Infusion was performed to treat critical hemorrhagic shock but was ineffective. We informed the patient and his family members of the situation, and once written informed consent to treatment was provided, we rushed him to the operating room.

**Diagnoses:**

Contrast medium permeated into the gastric cavity through a fistula between the abdominal aorta and gastric tube at the 11th thoracic level, Based on this, we made a diagnosis of AGF resulting from a peptic ulcer, and this diagnosis was further confirmed by high pressure angiography combined with computed tomography (CT) imaging.

**Interventions:**

An endovascular stent graft was placed under the guidance of digital subtraction angiography and followed by antibiotic therapy to prevent infection and proton pump inhibitor therapy to inhibit gastric acid secretion.

**Outcomes:**

The patient recovered uneventfully after the procedure. Four months after surgery, the patient died of organ failure caused by retroperitoneal lymph node metastasis and multiple intrahepatic metastases, with no postoperative bleeding linked to the endovascular stent graft repair.

**Lessons:**

Our case supports the notion that endovascular stent graft repair is a feasible alternative in treatment of AGF with several advantages in addition to surgical intervention, although more such cases should be collected and analyzed in the future to corroborate our observations.

## 1 Introduction

Aortogastric fistula (AGF), an abnormal communication between the aorta and the gastrointestinal tract, is a rare but fatal disorder after esophagectomy. Because the reconstructed gastric tube is adjacent to the major vessels and organs such as the heart, aorta, and trachea, ulceration-induced penetration of the gastric tube into any of these structures can be consequently serious and fatal.^[1,2]^ Surgical intervention has been typically used to treat AGF after esophagectomy, but mortality rates have been high. However, conservative treatment was always accompanied by a lethal outcome. Graft replacement appears to be a promising approach.^[2]^ Here, we report an unusual case of AGF that was effectively treated with placement of an endovascular stent graft. We obtained written informed consent from the patient to report this case.

## 2 Case presentation

**Figure 1:**
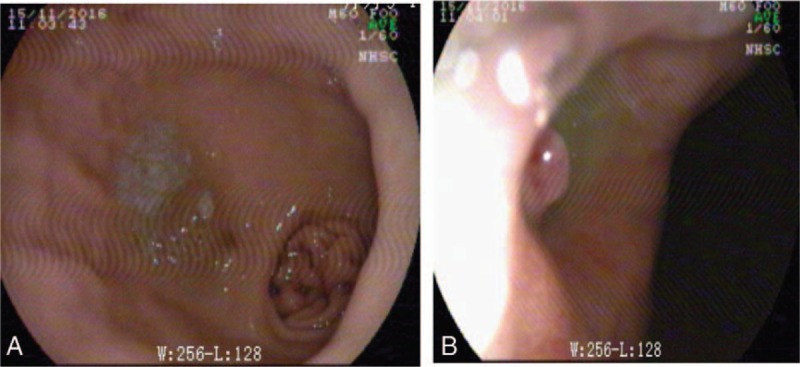
A peptic ulcer is observed by gastrointestinal endoscopy on the posterior wall of the distal part of the gastric tube.

A 59-year-old man with a history of type 2 diabetes and smoking (an average of 20 cigarettes per day) but no history of high blood pressure and heart disease underwent video-assisted thoracoscopic esophagectomy for squamous cell carcinoma (stage PT1N1Mx) of the distal esophagus with an esophageal reconstruction using a gastric tube through the posterior mediastinal route 14 months previously. The surgical resection margins were negative, and right lung nodule metastasis was not observed. The adjuvant radiotherapy (chemoradiotherapy for the original esophageal tumor area and lymphatic drainage area) and chemotherapy were completed. At 7 months postoperation, the patient occasionally experienced episodes of epigastric discomfort. A gastrointestinal endoscopic biopsy revealed a gastric ulcer on the posterior wall of the distal part of the gastric tube, but his vital signs were stable and routine blood, liver, and kidney function test results were within the normal ranges. This patient did not receive continuous treatment with a proton pump inhibitor. Five months previously, he experienced epigastric discomfort again and had blood in the stool. Gastrointestinal endoscopic examination revealed a round deep ulcer, 1.5 mm in diameter, in the gastric body, with visible blood vessels in the middle (Fig. 1A and B). However, there was no evidence of recurrence over the 1 year following the operation. Two days after the endoscopic examination, he suddenly experienced massive hematemesis and unstable circulatory dynamics, with a blood pressure of 90/45 mm Hg and a pulse of 150 beats/min. Blood tests revealed severe anemia, with hemoglobin (Hb) of 50 g/L and hematocrit of 15.3%. Infusion was performed to treat critical hemorrhagic shock but was ineffective. We informed the patient and his family members of the situation, and once written informed consent to treatment was provided, we rushed him to the operating room.

**Figure 2:**
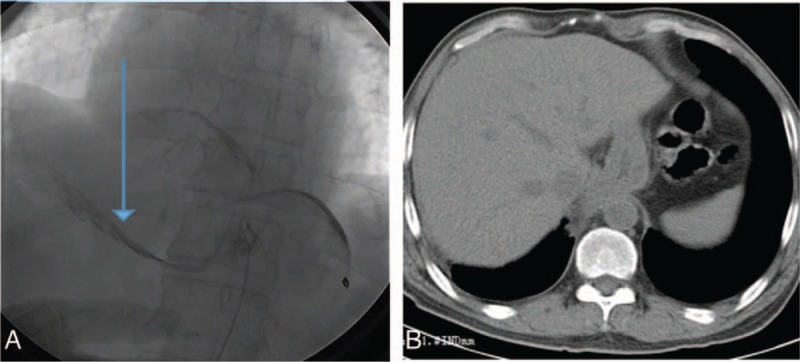
Emergency abdominal aortography (A) and CT (B) revealing remarkable extravasation of contrast medium that flowed out of the gastric tube. CT = computed tomography.

**Figure 3:**
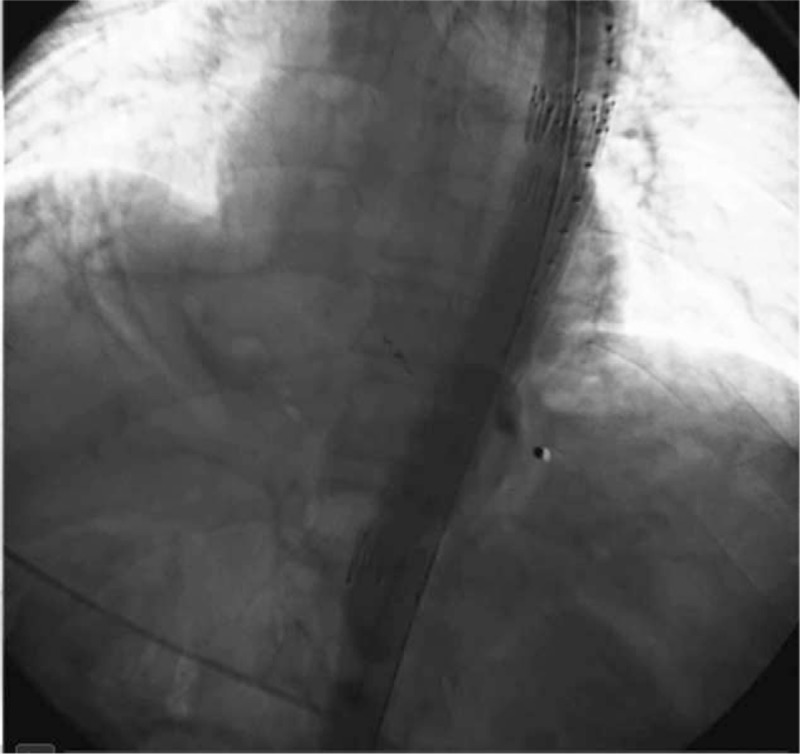
Aortography after stent graft placement around the fistula revealing hemostasis.

**Figure 4:**
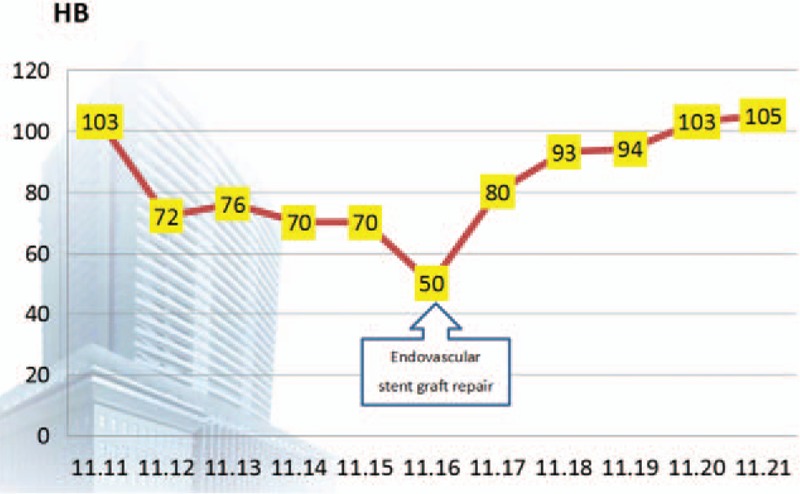
Changes in Hb levels before and after stent implantation in the patient. Hb = hemoglobin.

**Figure 5:**
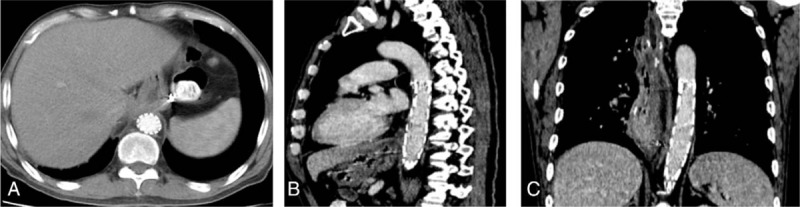
Contrast-enhanced CT showing no evidence of extravasation. CT = computed tomography.

Because of the patient's unstable circulatory dynamics, we classified him as high risk for open surgery and decided to use a stent graft repair as the first feasible option for urgent treatment. Diagnostic angiography and endovascular stent graft repair were performed under local anesthesia. After access was obtained via the femoral artery, a 4-Fr catheter was placed into the abdominal aorta, and we found that contrast medium permeated into the gastric cavity through a fistula between the abdominal aorta and gastric tube at the 11th thoracic level (Fig. 2). Based on this, we made a diagnosis of AGF resulting from a peptic ulcer, and this diagnosis was further confirmed by high pressure angiography combined with computed tomography (CT) imaging. We considered the AGF to be the primary cause of upper gastrointestinal bleeding. Thereafter, angiography was performed in the aortic arch by puncturing the left femoral artery and entering the marked pigtail tube, and the AGF was located. A stent (COOK ZTEG-2PT-32–160) was then passed through the right femoral artery into the aorta, positioned on the fistula location and released. After stenting, the graft was in good condition, no contrast medium was permeating into the stomach, and hematemesis ceased (Fig. 3). The patient's circulatory dynamics also stabilized immediately following the procedure, and he was then transferred to the intensive care unit (ICU) where he was kept for 1 day before being returned to the general ward (Fig. 4). The full procedure was performed under the guidance of digital subtraction angiography (DSA). Subsequent treatments included pumping norepinephrine, blood transfusion, infusion booster, sufentanil analgesia, and ceftriaxone. Also, omeprazole and octreotide were continuously administered to inhibit gastric acid secretion. The stent implantation, Hb concentration, and vital signs of this patient were relatively stable, and the patient did not have hematemesis, melena, or any other active bleeding. Considering that this patient had a tumor and was in a hypercoagulable state, in order to avoid thrombosis, he did not continue antibleeding treatment. At 2 months postoperation, in order to monitor the patency of the stent and active bleeding and related complications, we recommend that patients should have endoscopic review, but this patient and his family declined. Contrast-enhanced CT revealed no extravasation and no other complications such as hematemesis, infection, and pseudoaneurysm (Fig. 5). Four months after surgery, the patient died of organ failure caused by retroperitoneal lymph node metastasis and multiple intrahepatic metastases, with no postoperative bleeding linked to the endovascular stent graft repair.

## 3 Discussion

AGF is an uncommon but recognized life-threatening complication of esophagectomy. Anastomotic leakage and peptic ulcers are the 2 major causes for AGF after esophagectomy .^[2]^ There is an increasing number of reports of ulcerative lesions in reconstructed gastric tubes, which previously had been comparatively rare.^[1]^ Ide et al^[3]^ reported that the incidence of such lesions was 13%. The differences in the incidence rates reported by different studies can be attributed to the fact that an ulcer of the reconstructed gastric tube is often asymptomatic and thus goes unnoticed.^[4,5]^ If an ulceration is caused by penetration of the gastric tube into structures such as pericardium, aorta, and trachea, AGF can be serious and fatal.

Peptic ulcer formation has many etiologies including destruction of the mucosal barrier from ischemia in the gastric tube, hypersecretion of gastric acid, pulsation of the descending aorta that delays gastric emptying, *Helicobacter pylori* infection, and usage of nonsteroidal anti-inflammatory drugs (NSAIDs).^[6,7]^ The histological impact of postoperative radiotherapy has also been regarded as a significant contributor, as this treatment interferes with the histological responses necessary for healing.^[8]^ The above-mentioned factors, except *H pylori* infection, were present in our case. Moreover, our patient had a history of gastric ulcer. In addition, this patient had postoperative bleeding for 1 year and upper gastrointestinal angiography showed anastomotic patency.

Generally, no specific clinical manifestations are associated with the formation of an AGF after intrathoracic esophagogastric anastomosis,^[9]^ except some vague symptoms and signs including acute onset of mid-thoracic pain, heartburn, and sudden small volume or massive hematemesis. AGF should be highly suspected in patients having one of the aforementioned etiologies with at least one of those symptoms. Early accurate diagnosis is vital for survival. The most effective approach to diagnose an AGF is still CT scanning, which can reveal a number of characteristics such as the existence of novel nonhomogenous masses between the aorta and the stomach, air in the mediastinum and/or inside the aneurysm sac, as well as contrast extravasation from the aorta into the stomach.^[10]^ The definitive diagnosis can be established when ulcerations accompanied by coagulations are detected on endoscopy, although endoscopy-induced hypertension may increase the risk of further hemorrhage.^[11]^ In this particular case, the patient had mid-thoracic pain and sudden massive hematemesis, and an AGF was diagnosed by angiography in time.

In a recent report, nearly all AGF patients died of massive hemorrhage or aspiration of massive hematemesis.^[7,12–17]^ Therefore, timely appropriate treatment of AGF remains a challenge. The traditional management goals for AGF are the control of hemorrhage and sepsis and maintenance of lower limb perfusion. An aggressive surgical strategy consists of suturing the perforated aorta or aortic reconstruction via open surgery, which appears to be the sole management with appreciable long-term outcomes. Nevertheless, these surgical procedures have disadvantages including operation-linked stress and general anesthesia, which can exacerbate preexisting gastritis and stomach ulceration.^[13–19,20]^ Moreover, if the gastric tube is strongly adhered to the aorta around hiatus, an additional fenestration for the celiac artery would make the procedure even more complex and would prolong the operation time, thereby increasing the perioperative risk.^[16]^ Also, it was reported that pseudoaneurysms can develop after the operation.^[5]^

Recent studies reported successful treatments of AGF with endovascular stent graft repair.^[2,19]^ The advantages of using endovascular techniques include achieving rapid control of bleeding with minimal injury, avoiding intervention in a hostile abdomen, and eliminating the complications such as operative trauma and stress associated with open surgical repair^[19,21]^ The need for only local anesthesia is also one of the advantages. It was quite remarkable that our patient had a quick recovery and short hospital stay (∼2 weeks) compared with other successfully treated AGF cases in the literature.^[2,5]^ Regarding pseudoaneurysm recurrence, placement of an endovascular stent graft is also a useful backup modality if sterilization has been achieved.^[22]^ Considering the lethal course of this case, it should be stressed that celiac artery coverage needs to be considered very carefully, especially in patients with a known gastric ulcer as it may lead to a secondary AGF. One limitation of using endovascular stent graft repair is the high skill requirement for the practitioner. Another limitation of using this technique is that endovascular stent graft repair has been correlated with a high risk of infection, which is a factor contributing to poor outcome after endovascular repair.^[22]^ This also raises another concern of long-term safety and efficacy of this technique by placing a new prosthetic material in an already or potentially infected field. Thus, aggressive antibiotic therapy should be utilized following this procedure.^[23–25]^. In the present case, although there were no signs of infection, a prophylactic antibiotic therapy (i.e., ceftriaxone) was still prescribed because of the potential infection.

In this rare case, AGF was primarily attributed to a peptic ulceration. We performed angiography to achieve a correct diagnosis and treated the patient with an endovascular stent graft repair. We carried out the full procedure under the guidance of DSA, which allowed us to see the stent location clearly. Although the patient presented in a critical condition, the choice of the endovascular stent graft repair turned out to be very effective, as evidenced by the facts that no contrast leakage inside the stomach was observed and that the patient's hematemesis ceased as soon as the procedure was completed. During the disease progression, it is common for a gastric tube to continuously secrete acid even though the vagus nerve has been divided.^[26]^ Therefore, 24-hour pH surveillance of the gastric tube after an esophagectomy and a urease test should be carried out, and treatment to inhibit gastric acid production should be administered. In addition, endoscopic observation for the early diagnosis and appropriate management of AGF is also crucial for a favorable outcome. Previous studies have reported an interval between esophagogastrectomy and hematemesis in patients with peptic ulcers ranging from 14 months to 7 years.^[5]^ Hence, the best precautionary approach should be close follow-up of such patients during this period after esophagectomy. We considered the treatment of AGF in this patient with endovascular stent graft repair successful because the patient did not die from any events related to the endovascular stent graft repair but from cancer complications during the follow-up period.

In conclusion, we report here the use of endovascular stent graft repair to treat an unusual case of ulcer-linked AGF after esophagectomy. Our case supports the notion that endovascular stent graft repair is a feasible alternative in treatment of AGF with several advantages in addition to surgical intervention, although more such cases should be collected and analyzed in the future to corroborate our observations.

## References

[1] Mochizuki Y Akiyama S Koike M A peptic ulcer in a reconstructed perforating the thoracic aorta after esophageal replacement. Jpn J Thorac Cardiovasc Surg 2003;51:448–51. 14529164 10.1007/BF02719601

[2] Okamura A Kawakubo H Takeuchi H Successful treatment of aortogastric fistula after esophagectomy. Esophagus 2015;12:387–91.

[3] Ide H Eguchi R Nakamura T Late management of patients after esophagectomy and reconstruction for esophageal cancer (Eng abstr). Nihon Shokaki Geka Gakkai Zasshi 1995;28:2028–61.

[4] Katsoulis IE Veloudis G Exarchos D Perforation of a gastric tube peptic ulcer into the thoracic aorta. Dis Esophagus 2001;14:76–8. 11422314 10.1111/j.1442-2050.2001.00156.x

[5] Takebayashi T Okushiba S Ohno K Peptic ulcer-induced acute aortogastric fistula occurring 7 years after a pharyngogastrostomy following a resection for carcinoma of the esophagus: report of a case. Surg Today 2004;34:777–9. 15338354 10.1007/s00595-004-2826-1

[6] Takemura M Higashino M Osugi H Five cases of peptic ulcer of gastric tube after radical esophagectomy for esophageal carcinoma and analysis of *Helicobacter pylori* infection at gastric tube (in Japanese with English abstract). Nihon Kyoubugeka Gakkaizasshi (J Jpn Assn Thorac Surg) 1997;45:1992–7. 9455113

[7] Begin LR Sheiner NM Anastomotic ulcer-induced aortoenteric fistula after esophagogastroplasty. Ann Thorac Surg 1992;54:564–5. 1510529 10.1016/0003-4975(92)90458-g

[8] Hanashi T Ide H Nogami A A case report on the perforation of a gastric tube ulcer after esophageal reconstruction for cancer. Nippon Kyobu Geka Gakkai Zasshi 1991;39:1242–6. 1940534

[9] Kuhara A Koganemaru M Onitsuka S Emergent interventional approach for aortogastric tube fistula with massive gastrointestinal bleeding. BMJ Case Rep 2015;2015:pii: bcr2014208143. 10.1136/bcr-2014-208143 PMC4330418 25661750

[10] Chiesa R Melissano G Marone EM Aorto-oesophageal and aortobronchial fistula following thoracic endovascular aortic repair: a national survey. EurJ Vasc Endovasc Surg 2010;39:273–9. 20096612 10.1016/j.ejvs.2009.12.007

[11] Unosawa S Hata M Sezai A Surgical treatment of an aortoesophageal fistula caused by stent implantation for esophageal stenosis: report of a case. Surg Today 2008;38:62–4. 18085367 10.1007/s00595-007-3569-6

[12] Deutsch AA Reiss R Aortogastric fistula: an unusual complication of the thoracic portion of the stomach. Arch Surg 1978;113:537. 305774 10.1001/archsurg.1978.01370160195036

[13] Cowan JA Jr Dimick JB Wainess RM Ruptured thoracoabdominal aortic aneurysm treatment in the United States: 1988 to 1998. J Vasc Surg 2003;38:319–22. 12891114 10.1016/s0741-5214(03)00227-1

[14] Piazza M Ricotta JJ 2nd Open surgical repair of thoracoabdominal aortic aneurysms. Ann Vasc Surg 2012;26:600 e5. 22188939 10.1016/j.avsg.2011.11.002

[15] Gaudino M Lau C Munjal M Open repair of ruptured descending thoracic and thoracoabdominal aortic aneurysms. J Thorac Cardiovasc Surg 2015;150:814–23. 26227985 10.1016/j.jtcvs.2015.06.077

[16] Busuttil SJ Goldstone J Diagnosis and management of aortoenteric fistulas. Semin Vasc Surg 2001;14:302–11. 11740838 10.1053/svas.2001.27888

[17] Dorigo W Pulli R Azas L Early and long-term results of conventional surgical treatment of secondary aortoenteric fistula. Eur J Vasc Endovasc Surg 2003;26:512–8. 14532879 10.1016/s1078-5884(03)00379-4

[18] Leon LR Jr Mills JL Sr Jordan W The risks of celiac artery coverage during endoluminal repair of thoracic and thoracoabdominal aortic aneurysms. Vasc Endovascular Surg 2009;43:51–60. 18996912 10.1177/1538574408322655

[19] Heidemann F Diener H Debus S Repair of a contained ruptured paravisceral aortic aneurysm using a surgeon-modified fenestrated endograft and development of an aortogastric fistula. Ann Vasc Surg 2016;36:e294.e13–7. 10.1016/j.avsg.2016.03.023 27423722

[20] Kougias P Baltazar U Battle WJ Primary aortogastric fistula after nissen fundoplication: a case report and review of pertinent literature. Vasc Endovascular Surg 2003;37:135–9. 12669146 10.1177/153857440303700209

[21] Antoniou AG Koutsias S Antoniou SA Outcome after endovascular stent graft repair of aortoenteric fistula: a systematic review. J Vasc Surg 2009;49:782–9. 19028054 10.1016/j.jvs.2008.08.068

[22] Sato O Miyata T Matsubara T Successful surgical treatment of aortogastric fistula after an esophagectomy and subsequent endovascular graft placement: report of a case. Jpn J Surg 1999;29:431–4. 10.1007/BF02483034 10333413

[23] Chiesa R Tshomba Y Kahlberg A Management of thoracic endograft infection. J Cardiovasc Surg (Torino) 2010;51:15–31. 20081759

[24] Sato O Miyata T Matsubara T Successful surgical treatment of aortogastric fistula after an esophagectomy and subsequent endovascular graft placement: report of a case. Surg Today 1999;29:431–4. 10333413 10.1007/BF02483034

[25] Takano S Katsuhara K Nobuhara K Aortoesophageal fistula due to esophageal ulcer. Gen Thorac Cardiovasc Surg 2009;57:255–7. 19440823 10.1007/s11748-008-0378-9

[26] Rasuli P Hammond DI Eidus LB Peptic ulcer induced gastroaortic fistula: difficulty in making the diagnosis with selective angiography. Can Assoc Radiol J 1990;41:151–2. 2354390

